# Toward uniform implementation of parametric map Digital Imaging and Communication in Medicine standard in multisite quantitative diffusion imaging studies

**DOI:** 10.1117/1.JMI.5.1.011006

**Published:** 2017-10-30

**Authors:** Dariya Malyarenko, Andriy Fedorov, Laura Bell, Melissa Prah, Stefanie Hectors, Lori Arlinghaus, Mark Muzi, Meiyappan Solaiyappan, Michael A. Jacobs, Maggie Fung, Amita Shukla-Dave, Kevin McManus, Michael Boss, Bachir Taouli, Thomas E. Yankeelov, Christopher Chad Quarles, Kathleen Schmainda, Thomas L. Chenevert, David C. Newitt

**Affiliations:** a University of Michigan, Radiology, Ann Arbor, Michigan, United States; b Brigham and Women’s Hospital and Harvard Medical School, Boston, Massachusetts, United States; c Barrow Neurological Institute, Division of Imaging Research, Phoenix, Arizona, United States; d Medical College of Wisconsin, Radiology Research, Milwaukee, Wisconsin, United States; e Translational and Molecular Imaging Institute, Icahn School of Medicine at Mount Sinai, New York, New York, United States; f Vanderbilt University Medical Center, Institute of Imaging Science, Nashville, Tennessee, United States; g University of Washington, Imaging Research Laboratory, Seattle, Washington, United States; h Johns Hopkins School of Medicine, The Russell H. Morgan Department of Radiology and Radiological Science and Sidney Kimmel Comprehensive Cancer Center, Baltimore, Maryland, United States; i Memorial Sloan Kettering Cancer Center, GE Healthcare, New York, Unites States; j Memorial Sloan Kettering Cancer Center, Departments of Medical Physics and Radiology, New York, New York, United States; k University of Colorado Boulder, Department of Physics, Boulder, Colorado, United States; l National Institute of Standards and Technology, Applied Physics Division, Boulder, Colorado, United States; m University of Texas at Austin, Biomedical Imaging, Austin, Texas, United States; n University of California San Francisco, Radiology and Biomedical Imaging, San Francisco, California, United States

**Keywords:** quantitative diffusion-weighted imaging, apparent diffusion coefficient, parametric map Digital Imaging and Communication in Medicine, multisite trials

## Abstract

This paper reports on results of a multisite collaborative project launched by the MRI subgroup of Quantitative Imaging Network to assess current capability and provide future guidelines for generating a standard parametric diffusion map Digital Imaging and Communication in Medicine (DICOM) in clinical trials that utilize quantitative diffusion-weighted imaging (DWI). Participating sites used a multivendor DWI DICOM dataset of a single phantom to generate parametric maps (PMs) of the apparent diffusion coefficient (ADC) based on two models. The results were evaluated for numerical consistency among models and true phantom ADC values, as well as for consistency of metadata with attributes required by the DICOM standards. This analysis identified missing metadata descriptive of the sources for detected numerical discrepancies among ADC models. Instead of the DICOM PM object, all sites stored ADC maps as DICOM MR objects, generally lacking designated attributes and coded terms for quantitative DWI modeling. Source-image reference, model parameters, ADC units and scale, deemed important for numerical consistency, were either missing or stored using nonstandard conventions. Guided by the identified limitations, the DICOM PM standard has been amended to include coded terms for the relevant diffusion models. Open-source software has been developed to support conversion of site-specific formats into the standard representation.

## 1 Introduction

There is growing interest across the clinical trial community^1^^–^^4^ to utilize quantitative diffusion metrics^5^^–^^8^ for imaging assessment of oncology patients.^9^^–^^11^ The most frequently utilized quantitative (isotropic) diffusivity metrics is apparent diffusion coefficient (ADC), which reflects mean water mobility known to be sensitive to cellular constituents within tissue.^12^^,^^13^ For parametric maps (PMs) of imaged volume, the ADC values are derived from two or more diffusion-weighted imaging (DWI) measurements assuming mono-exponential signal decay with increasing diffusion weighting, b-value. Organ-specific clinical trial protocols using ADC measurements are being incorporated into ongoing studies for early evaluation of treatment efficacy.^9^^,^^10^^,^^14^^–^^16^ Utilization of quantitative diffusion parameters for multisite, multiplatform oncology trials requires standardization of both DWI acquisition protocols and analysis software for reduction of technical variability and establishment of robust imaging biomarkers.^17^^–^^19^ To monitor protocol compliance for multisite DWI acquisitions and to facilitate centralized meta-analysis of multimodel diffusion metrics, both DWI and parametric diffusion map metadata need to be captured and stored in standard formats.

The widely accepted imaging data standard for annotation and storage across the clinical imaging community is the Digital Imaging and Communication in Medicine (DICOM) standard.^20^ Relevant to the standardized representation of diffusion MRI, DICOM provides structures, coded terms, and attributes that can be used to describe DWI acquisition in the enhanced multiframe MR image object.^21^ However, frequently MR manufacturers are generating DWI DICOM outputs that are stored in nonenhanced objects and use nonstandard storage attributes for DWI acquisition parameters. The lack of standard-prescribed mechanisms for communicating critical DWI-related attributes (e.g., b-value and directionality) in these objects results in challenges for consistent interpretation of the DWI data and its analysis in multicenter, multiplatform studies. Furthermore, the storage of parametric diffusion maps generated on MR scanners or by postprocessing tools is also not standardized across the platforms, limiting reliability of derived quantitative metrics both for research and clinical applications.

To address these hurdles in communicating quantitative diffusion parameters, the DICOM PM object^22^ was introduced for the discussion of the DICOM community in Supplement 172^23^ and was subsequently integrated into the standard in the 2014b revision. In general, the term “PM” is used to refer to any derived image whose pixel values have a quantitative meaning, as opposed to acquired (or derived) images whose pixel values may be arbitrary signal intensities. The work on developing the PM object was initiated by the Quantitative Image Informatics for Cancer Research (QIICR) project^24^ in order to address limitations of the existing storage objects. First, the DICOM attributes that are needed to describe important aspects of the PMs (e.g., quantity being represented and units of the pixel values) were not well-defined, and the standard codes were missing, leading to the challenges of consistent machine-readable representations of PM objects. Second, it was not possible to include floating-point pixel data, which is sometimes required to properly represent the results of image analysis. These limitations were addressed in the DICOM PM storage object. Furthermore, the conversion from the popular research formats to DICOM PM has recently been implemented in the *dcmqi* (DICOM for Quantitative Imaging) library.^25^ A distinguishing characteristic of a parametric ADC map is that each pixel value relates to a physical quantity derived from a specific diffusion model using DWI acquisition parameters. Therefore, the DICOM PM object that stores the resulting ADC data should capture relevant metadata adequately describing both acquisition and fit model for complete representation.

The goal of this collaborative project was to review the current state of support for DICOM-based storage of parametric diffusion maps in the National Cancer Institute (NCI) Quantitative Imaging Network (QIN)^26^ community and, accordingly, evaluate the capabilities of the DICOM PM storage for DWI-specific applications and models. The results are intended to guide the development of standard-compliant conversion tools to facilitate wider and more consistent implementation across the quantitative diffusion imaging community. This effort was complimentary to the ongoing QIN-wide ADC mapping challenge^27^ that suffered from the lack of standard output format for quantitative diffusion analysis across sites. In the context of centralized meta-analysis for multicenter oncology trials, the DICOM PM allows for the capture of metadata critical to the interpretation of the ADC results. This facilitates identification and elimination of nonbiological variability in the derived quantitative diffusion values. To establish these critical metadata attributes, this collaborative study was designed to evaluate both numerical consistency, with respect to ground-truth diffusion values known for a phantom, and the ability of the DICOM metadata stored by individual analysis software to reflect specific sources of detected numerical discrepancies in ADC values.

## 2 Methods

Ten participating sites were provided with a single multivendor DWI DICOM dataset for a quantitative DWI phantom (HPD Inc., Boulder, Colorado) and instructed to generate parametric ADC maps using a mono-exponential fit for DWI signals acquired with four b-values (ADC4) and the two (b=0, highest-b) b-value pair (ADC2). To imitate a multicenter, multiplatform analysis environment, the sites were expected to discover the corresponding b-values and vendor-specific image order from the supplied DWI DICOM header. The resulting ADC2 and ADC4 DICOM objects were evaluated for numerical consistency between the two fit models and among site algorithms by a central analysis lab (University of Michigan) to identify ADC discrepancies directly stemming from fit models and parameters. The ADC DICOM metadata was then evaluated for the ability to properly reflect the sources of detected numerical discrepancies and for presence of common attributes suggested by the PM standard document.^23^ All ADC DICOM site results were uploaded to project M-box (hosted by SW5 site) for centralized analysis. The cross-site analysis was performed in MATLAB^®^ 7 R2015b (MathWorks, Natick, Massachusetts), using the built-in *statistics* and *imaging* toolbox functions.

### 2.1 Phantom DWI DICOM

A quantitative DWI phantom developed by the National Institute of Standards and Technology (NIST)^28^ provided a linear range of one-order-of-magnitude in ADC (±0.005): $(0.125,0.24,0.40,0.60,0.83,1.10)\times 10^{-3}\;{\mathrm{mm}}^{2}/s$ (at 0°C, “ground-truth” values based on multisite studies).^29^^,^^30^ This allowed simultaneous evaluation of fit accuracy over a broad range of DWI intensities in a single image. Briefly, the phantom contained two vials for each of five polyvinylpyrrolidone (PVP) water solutions (10% to 50%) and three vials with pure water (for a total of 13 vials) arranged hexagonally within the spherical phantom shell. For temperature control, the shell was filled with an ice-water bath. During scanning, the central water vial was positioned close to the magnet isocenter, and other vials were radially offset up to 55 mm from the central vial. The phantom DWI data were acquired at 0°C on Siemens Trio (S1), GEMS Discovery MR750 (S2), and Philips Ingenia (S3) 3T scanners for five coronal slices using b-values=0, 500, 900, and $2000\;s/{\mathrm{mm}}^{2}$ and following a common scan protocol.^18^

Trace-DWI DICOM for these multivendor phantom scans was shared with the QIN MRI subgroup by Quantitative Imaging Biomarker Alliance (QIBA) DWI task force members. Full scanner-generated headers were provided in the single-frame format (DICOM MR Storage object) without deidentification. “S3” DWI DICOM was supplied in multiframe (DICOM enhanced MR storage object) and single-frame format. “S1” and “S2” scanners were not capable of generating multiframe DWI DICOM.^21^ Vendor-specific DICOM conformance documents were shared with the group through NCIP-HUB.^31^ Two vendors stored the diffusion b-value and associated directionality information in nonstandard attributes [S1: (0019, 100C/D) and S2: (0043, 1039/30)]. The information on vendor-specific image order and b-value storage attribute was requested by users of SW1 (S3), SW7 (S3), and SW8 (S1 and S3), while others were able to parse multivendor source DWI DICOM. Three sites (SW3, SW7, and SW10) analyzed multiframe DWI DICOM from the “S3” source.

### 2.2 ADC Map DICOM

The data inventory for ADC maps submitted by sites is summarized in Table 1. Ten sites generated parametric ADC2 and eight sites provided ADC4 mono-exponential diffusion model fits. All sites performed ADC2 fit using a log-intensity ratio to high b-value. Different multi-b fit algorithms reported by sites for ADC4 maps are listed in Table 1. SW1 and SW5 were fitting “unweighted” log-intensity DWI allowing extra fit parameter ($S_{0}$, DWI intensity at b=0). SW2 SW3 used a combination least-squares regression algorithm for log-intensities, which effectively “weights” signals by the average DWI intensity (that generally depends on the range of the applied b-values). SW2, SW4, SW6, SW7, and SW10 performed regression or least-squares fit for log-signal intensities that effectively “weights” DWI by signal-to-noise ratio (SNR). Five sites used third-party software (two on-scanners and three offline) to generate ADC maps, and five sites have utilized their in-house tools for offline analysis. According to the site reports, only one vendor SW8 was capable of cross-vendor DWI analysis off-scanner to generate ADC2 (ADC4 allowed, but not generated), and it required manual entry of b-value and DWI image order for S1 and S3 data. SW8 also produced interpolated ADC maps for S1 (256×256) and S3 (512×512) images. All other maps preserved source-image dimensionality. One vendor SW (SW10) provided both ADC2 and ADC4 maps for its DWI data source (S3).

**Table 1:** Details of ADC analysis software (SW) and algorithms used in the study.

Label^i^	Name (source)	Version	ADC4 fit (*function*)^ii^	DWI processediii	ADC2/ADC4	Scale ($10^{-3}\;{\mathrm{mm}}^{2}/s$)
SW1^(c)^	MATLAB^®^ (site)	R2015b	LLS ($S_{0}$) (*poly-fit*)	S1, S2, S3	y/y	$∼10^{4}$
SW2^(h)^	MapMaker (C++, site)	1.0	LLS (*regression*)	S1, S2, S3	y/y	$10^{3}$
SW3^(d)^	IB diffusion (IB LLC^iv^)	2.0.104	LLS (*lsq-regression*)	S1, S2, S3	y/y	$∼10^{4}$
SW4^(e)^	MATLAB^®^ (site)	R2015b	LLS (*lsq-fit*)	S1, S2, S3	y/y	$10^{3}$
SW5^(n)^	Adcmap (IDL, site)	3.0	NLS ($S_{0}$) (*curve-fit*)	S1, S2, S3	y/y	$10^{3}$
SW6^(a)^	QIBAPhan (QIDW^v^)	R1.2	LLS (*lsq-fit*)	S1, S2, S3	y/y	$10^{4}$
SW7^(g)^	ADCmap (OsiriX^vi^)	1.9	LLS (na)	S1, S2, S3	y/y	$10^{3}$
SW8^(i,j)^	ReadyView (GEvii)	14.3.0	${\mathrm{ND}}^{+}$ (*lsq-fit*)	S1, S2, S3	$y/n^{+}$	$10^{3}$
SW9^(k,l)^	SyngoMR (Siemens)	B17	NA	S1	y/n	$10^{3}$
SW10^(f)^	Achieva3T (Philips)	5.1.7	LLS (*lsq-fit*)	S3	y/y	$∼10^{4}$

i SW label superscript indicates institution of origin corresponding to the affiliations in the author list.

ii ADC2 fit not listed, since it was performed by all sites using log-intensity difference ratio to high b-value. LLS, linear least squares; NLS, nonlinear least squares; $S_{0}$, extra fit parameter; NA, info not available; ${\mathrm{ND}}^{+}$, data not submitted [LLS (*lsq-fit*) available, according to vendor]; *lsq*, least squares; and *poly*, polynomial.

iii DWI source scanner labels S1: Siemens Trio; S2: GE Discovery MR750; and S3: Philips Ingenia.

iv Imaging Biometrics LLC, Elm Grove, Wisconsin.

v Quantitative Imaging Data Warehouse, RSNA.^32^

vi OsiriX ADCMap plugin.^33^

vii Ref. 34.

For numerical consistency and header checks, ADC DICOM data provided by each of the sites were loaded into MATLAB^®^ using standard *dicomread* function. The image scaling recorded in the header [(0040, 9096) “RealWorldValueMapping” or (0028, 1053) “RescaleSlope”] was used to convert all maps to the common scale of $10^{4}$ integer intensity range ($10^{-7}\;{\mathrm{mm}}^{2}/s$) before cross-site analysis. SW2 provided incorrect scaling for S3 data, apparently carried over from the source DWI DICOM header, which was ignored. SW2, SW4, and SW7 implementations did not record scale information (tag missing or value set to 1), and apparent integer map scale was used instead (see Table 1). SW1 provided scale value with inverse interpretation, which was likewise corrected. No additional filtering or masking was performed for the site ADC maps.

### 2.3 Numerical Consistency

The purpose of the numerical consistency analysis was (1) to determine a consensus ADC value range in relation to adequate scale and bit storage for the PM presentation and (2) to relate deviations between ADC4 and ADC2 fit algorithm results, with respect to the ground-truth values provided by the phantom, to the metadata required to describe observed discrepancies.

**Fig. 1:**
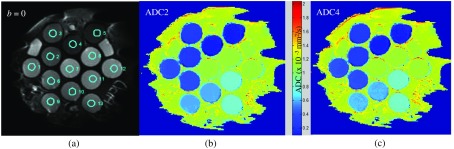
(a) illustrates example screen-captures of a middle slice for T2-weighted image (with inscribed, numbered ROIs) for the PVP phantom (scanner 1 source, S1) and the corresponding (b) ADC2 and (c) ADC4 maps generated by “SW3” analysis. Two ROIs are defined for each %PVP>0 concentration and three for ice-water (%PVP=0). Common scale for the ADC2 and ADC4 maps is indicated by the color-bar between (b) and (c).

Apparent, slightly different placement of the phantoms in different scanners necessitated defining separate (source-specific) sets of regions-of-interest (ROIs) for statistical analysis of the ADC results. ADC ROI statistics were generated using the *qiba_proc* function of QIBAPhan software (Table 1). Briefly, a common set of 13 circular ROIs (1-cm diameter) was defined on PVP vials from a middle (b=0) slice image [Fig. 1(a)] for each data source (S1, S2, and S3) and automatically applied to the ADC maps [e.g., Figs. 1(b) and 1(c)] provided by participating sites to generate mean, median, standard deviation (SD), and ranges that were recorded in coma-separated-value tables along with the ROI coordinates. Location of the central tube ROIs varied among source data from 15 to 30 mm (from isocenter). The difference in locations of duplicate ${\mathrm{ADC}}_{\mathrm{PVP}}$ ROIs with respect to isocenter was from 25 to 50 mm. Consequently, up to 3% systematic variations were expected for mean ADC values between duplicate PVP ROIs of different DWI sources due to variability of source scanner gradient characteristics.^35^

Numerical consistency of mean ADC2 versus ADC4 with respect to the ${\mathrm{ADC}}_{\mathrm{PVP}}$ reference was analyzed by scatter plots for 13 ROIs for all data sources within order of magnitude range provided by the PVP phantom. The coefficient of variance (CV) for ADC ROIs was calculated as ${\mathrm{SD}}_{\mathrm{ROI}}/{\mathrm{ADC}}_{\mathrm{PVP}}$. The Bland–Altman test was performed for the SW1 to SW7 data that provided results from both fit algorithms for all three DWI data sources. Deviation of mean ADC4 from ADC2 results supplied metrics for evaluation of the fidelity of the multi-b fit algorithm. The source of deviations between ADC4 and ADC2 was tracked back to the specific fit algorithm and parameters (Table 1) to determine relevant descriptive metadata.

### 2.4 DICOM Implementation Comparison to the PM Standard

Site ADC DICOM implementations were compared to the general PM^23^ and DWI DICOM macro requirements.^21^ The required attributes and coded terms of the PM DICOM metadata were defined in the DICOM PM storage object.^23^ These included image type, storage format, image scale and units, image geometry, and DWI source-image reference. Additional attributes important for numerical consistency analysis specific to the quantitative DWI PM, but not present in the original standard, included fit algorithm and parameters (i.e., the b-values used). The DICOM header evaluation was performed using the MATLAB^®^ structure query functions and a DICOM validator command-line application (*dciodvfy*).^36^ To check compatibility with existing DICOM parsers/viewers, the ADC maps submitted by sites were loaded into a production-level viewer, iQ-View 2.8.0 (Image Information Systems, UK), for the clinical picture archiving and communicating system (PACS), as well as into five additional DICOM viewers widely utilized by the imaging research community [OsiriX 8.0.2 (Pixmeo, Switzerland), IDL 8.5 (Excelis, Boulder, Colorado), 3D Slicer 4.7.0 (NA-MIC, NIH), ImageJ 1.45s (NIH), and KPacs 1.6.0 (Image Information Systems, UK)]. Viewing of ADC DICOM for SW1, SW4, and SW6 (S2 and S3) required resetting window-level at full dynamic range.

The requirements regarding storage of the DWI model and fitting-related parameter deficiencies were used to develop a correction proposal^37^ to improve the DICOM standard for quantitative analysis of DWI and to facilitate uniform implementation in the future by the QIN community through development of the open-source DICOM for Quantitative Imaging (*dcmqi*) library^25^^,^^38^ of converter tools. This converter can be applied to the volumetric image data stored in any of the supported formats (e.g., MHD,^39^ NifTi,^40^ and Analyze^41^) and can construct a DICOM PM header by merging source DWI DICOM with optional metadata supplied by the user in an ASCII (JSON)^41^ file. The application details are discussed in the *dcmqi* user guide.^42^ The converter was tested by a central analysis lab (SW6) on Win64 platform. Briefly, an S2 ADC2 volumetric image dataset was converted to MHD (one of the allowed input formats) and an example JSON metadata file was edited to include units, b-value and model used. The converter was applied to volumetric MHD ADC, using the corresponding source DWI DICOM and metadata. The resulting DICOM PM object header was queried in MATLAB^®^ using *dicominfo* function and tested for the import to iQ-View, OsiriX, IDL, 3D Slicer (with and without the *QuantitativeReporting* extension), ImageJ, and KPacs.

## 3 Results

Out of 10 project participants, 7 generated complete datasets for both ADC2 and ADC4 and all (three) DWI sources. The main challenges faced by the participants with the source multivendor DWI DICOM data were related to (1) parsing the dimensionality of the DWI acquisition first by the b-value and then by the geometric position of the individual image frames and (2) parsing the b-values that were in most cases stored in nonstandard DICOM tags. The sites that used on-scanner SW have confirmed that multivendor DWI DICOM could not be “parsed” across vendors. Out of five sites using third-party offline tools, three requested additional info for parsing the DWI DICOM header to extract b-values. Interestingly, this additional info was requested for the S3 data source that implements standard DWI DICOM tags. ADC2 and ADC4 maps generated from S3 multiframe DWI DICOM by three sites were identical to single-frame maps.

### 3.1 Numerical Consistency of ADC2 versus ADC4 across Site Implementations

**Fig. 2:**
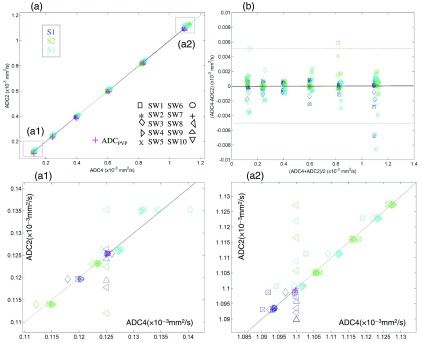
Panel (a) shows a scatter plot of ROI-mean ADC2 versus ADC4 values for all SWs and ROIs. Magenta “+” marks true ${\mathrm{ADC}}_{\mathrm{PVP}}$ values. Dotted diagonal line marks ADC2 = ADC4. Legends list symbol assignment for site software (SW) and color-code for data sources (S1, S2, and S3). The ROI clusters corresponding to the lowest ($0.125\times 10^{-3}\;{\mathrm{mm}}^{2}/s$) and the highest ($1.1\times 10^{-3}\;{\mathrm{mm}}^{2}/s$) ${\mathrm{ADC}}_{\mathrm{PVP}}$ values are enlarged in panels (a1) and (a2), respectively. Vertically aligned symbols correspond to ADC2 data submitted without ADC4 maps. Panel (b) shows Bland–Altman plot for ADC4 versus ADC2 generated by the seven sites that submitted all required DICOM for all data sources. Dotted horizontal lines mark 98th data percentile.

Figure 2 shows high numerical consistency of mean ROI ADC values across sampled SWs. All measurements are tightly clustered around six values resolving discrete ADC contrast levels in Figs. 1(b) and 1(c), corresponding to different PVP concentrations. This confirms correct interpretation of the ADC map scaling information (Sec. 2.2). The clusters are slightly offset, depending on data source (color-coded), as ADC increases, consistent with the expected systematic differences between scanners gradient systems and ROI locations (see Sec. 2.3). A closer look at the lowest and highest mean ADC values (Fig. 2, panels a1 and a2) shows further clustering for each data source corresponding to two and three ROIs per PVP concentration, for low and high ADC, respectively. ROI cluster proximity to the true ${\mathrm{ADC}}_{\mathrm{PVP}}$ value (marked by magenta “+”) is evidently determined by ROI location and data source (within 2% for high and 8% for low ADC) and is nominally independent of the analysis SW. For specific ${\mathrm{ADC}}_{\mathrm{PVP}}$ value, the ADC ROI CV (data, not shown) depended mainly on the data source (independent of ROI location) and decreased with growing ${\mathrm{ADC}}_{\mathrm{PVP}}$ from 10% (a1) to 0.05% (a2), indicative of the contrast-to-noise ratio (CNR) limits for the low ${\mathrm{ADC}}_{\mathrm{PVP}}$.

Panels a1 and a2 from Fig. 2 show enlargements of the highest and lowest ADC “clusters” illustrating that systematic mean ADC deviations among data sources (color-coded) and ROIs exceed the differences among analysis SW [symbol legend, Fig. 2(a)] along both the ADC2 (vertical) and ADC4 (horizontal) dimensions. For each individual ROI cluster, the spread is higher along ADC4 compared to ADC2, indicating better consistency of the ADC2 fit across sites. Note that for the on-scanner SW that provided only ADC2 maps (vertically aligned symbols), the deviation from the centers of the ROI clusters is usually higher than among the other offline tools (Table 1). This is likely related to additional signal interpolation and filtering applied by the scanner SW in contrast to offline processing.

The apparent increase in scatter along the ADC4 dimension for some ROI clusters (duplicate PVP concentrations in panels a1 and a2 from Fig. 2) is mainly due to the presence of three “outliers” (SW1, SW3, and SW5) that happen to use different multi-b fit functions (signal “weighting”) compared to the other sites (Table 1, Sec. 2.2). For the sites that submitted both ADC2 and ADC4 maps, the differences between mean ADC4 and ADC2 for each SW are shown as a function of the average measured ADC [Fig. 2(b)]. When compared to the true phantom ${\mathrm{ADC}}_{\mathrm{PVP}}$, these differences can be used as a self-normalized measure of algorithm fidelity for the mono-exponential diffusion medium provided by the phantom. For the majority of data, the observed ADC4 versus ADC2 deviations are within $\pm 0.002\times 10^{-3}\;{\mathrm{mm}}^{2}/s$ and nearly independent of the ADC and ROI location, except for the above-mentioned three ADC4 fit algorithm “outliers.” The outlier deviations are higher (and exceed ROI CV) at higher ${\mathrm{ADC}}_{\mathrm{PVP}}$, suggesting that they are related to SNR bias for high b-values when all DWI signals (including b=0) are given equal weight in the fit (Table 1, Sec. 2.2). The largest relative deviations observed for SW1 and SW5 at high ${\mathrm{ADC}}_{\mathrm{PVP}}$ are likely related to the use of an extra (intercept) parameter by their corresponding ADC4 fit functions (Table 1, Sec. 2.2) compared to the slope-only calculation by ADC2 and b-value-dependent weighting used for ADC4 by SW2, SW4 to SW7. The detected variations among fit results are clearly significant enough to warrant inclusion of the corresponding descriptive metadata (b-values used and fit functions/parameters) in the generated ADC map DICOM header.

### 3.2 Site DICOM Implementation versus Parametric Map Standard

**Fig. 3:**
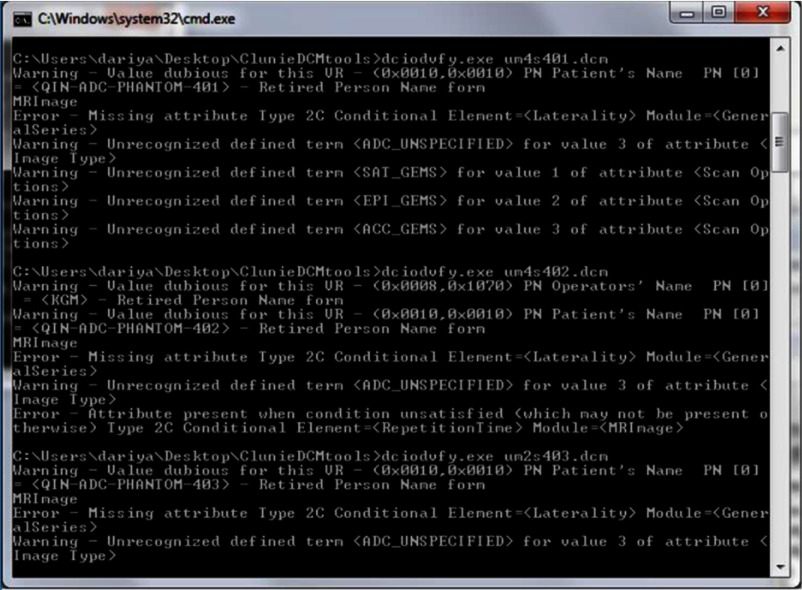
Screen-capture of *dciodvfy* check results for multivendor ADC DICOM generated by SW6.

Table 2 summarizes the results of ADC DICOM header evaluation across sites. All sites generated single-frame ADC maps and used in-house variations of the DICOM MR object for ADC map storage. Except for the SW2 that copied the source DWI DICOM header, coded terms for the site ADC DICOM implementations were supplied by OsiriX, MATLAB^®^, IDL, and scanner DICOM dictionaries (Table 2, source applications). Source DWI geometry was preserved by most implementations. SW4, SW6, and SW7 reversed the slice order for S3, S2, and S2, respectively, while SW2 had wrong slice locations assigned to S3 ADC, which were manually corrected. Except for SW4 (32-bit maps), all ADC map DICOMs were successfully parsed and imported to iQ-View production PACS viewer, as well as, to five additional tested parsers/viewers (listed in Sec. 2.4), indicating general back-compatibility. All headers passed the DICOM validator test with “warnings” for nonstandard attributes and lookup tables, and “errors” related to missing conditional attributes (e.g., Appendix, Fig. 3).

**Table 2:** ADC DICOM attributes in site implementations.

SW label	DCM source application	Storage format	Scale tag^a^	Unit tag	Source DWI ref.	b-value info	Fit algo. info	Image type “ADC”
SW1	OsiriX	Int15	RSI	NA	NA	NA	NPI	n
SW2	Source DWI	Uint16, Int16 (S2)	RS=1	NA	NA	NA	NA	n
SW3	OsiriX	Uint16	RS	NA	NA	NA	NPI	n
SW4	MATLAB^®^	Uint32	NA	NA	NA	NA	NPI	n
SW5	IDL	Uint16	NS	NS	NS	NS	NS	y
SW6	MATLAB^®^	Uint16	NS	NS	NS	NS	NS	y
SW7	OsiriX	Uint16	RS=1	RT=US	NA	NA	NA	n
SW8	ReadyView	Int16	RW	RW	NA	NA	NA	y
SW9	SyngoMR	Uint12	NS	NA	NA	NA	NA	y
SW10	Achieva3T	Uint12	RS	RT	NA	NA	NA	y

a RS, rescale slope; RS=1, not used; RSI, nonstandard value interpretation; RW, RealWorldValue; NS, nonstandard; NA, no attribute; RT, RescaleType; RT=US, not used; and NPI, information not provided.

None of the project participants stored the analysis results using the DICOM PM object. SW8 ADC DICOM contained the most common attributes with the DICOM PM (including standard attributes for ADC units and scale.) ADC fit parameters (b-values) and models (algorithms), source-image reference and ADC units/scale attributes were missing from most site implementations or stored in nonstandard (e.g., comment or private) fields (Table 2). Missing or nonstandard DICOM tags for units and scale attributes complicated meta-analysis and numerical interpretations (see Sec. 2.2).

The ADC map scaling consensus across all sites was in the range of ($10^{3}$ to $10^{4}$) (Table 1), suggesting that 16-bit precision was found sufficient for ADC map storage. The actual bit-depth stored in site DICOMs exceeded the sufficient range and varied across the site SW tools [from uint12 to u/int16 for most, and uint32 (for SW4), Table 1]. SW1, SW2(S3), and SW8 have used “signed” integer data storage, which allowed unphysical (negative) ADC values. Only one SW4 has utilized 32-bit (integer precision) DICOM storage of comparable range to that offered by PM standard. However, this broad range apparently accommodated background noise values (outside of the observed ADC range).

In the course of the project, the need for coded terms to accommodate a DWI-specific fit algorithm and parameters resulted in preparation of a correction proposal, and the DICOM standard was extended to include the missing attributes. Based on the recently amended standard, multiplatform (PC, Mac, and Linux) open-source *dcmqi* command-line converter tools have been developed by QIICR to generate 32-bit floating-point multiframe ADC DICOM PM object for several commonly used input formats (NIfTi, Analyze, MHD, etc.). The tools automatically extract relevant source DWI DICOM header information and allow (manual) addition of optional (text) metadata input, including units, b-values, and diffusion model, to annotate volumetric ADC images, preconverted to the allowed input formats.

**Fig. 4:**
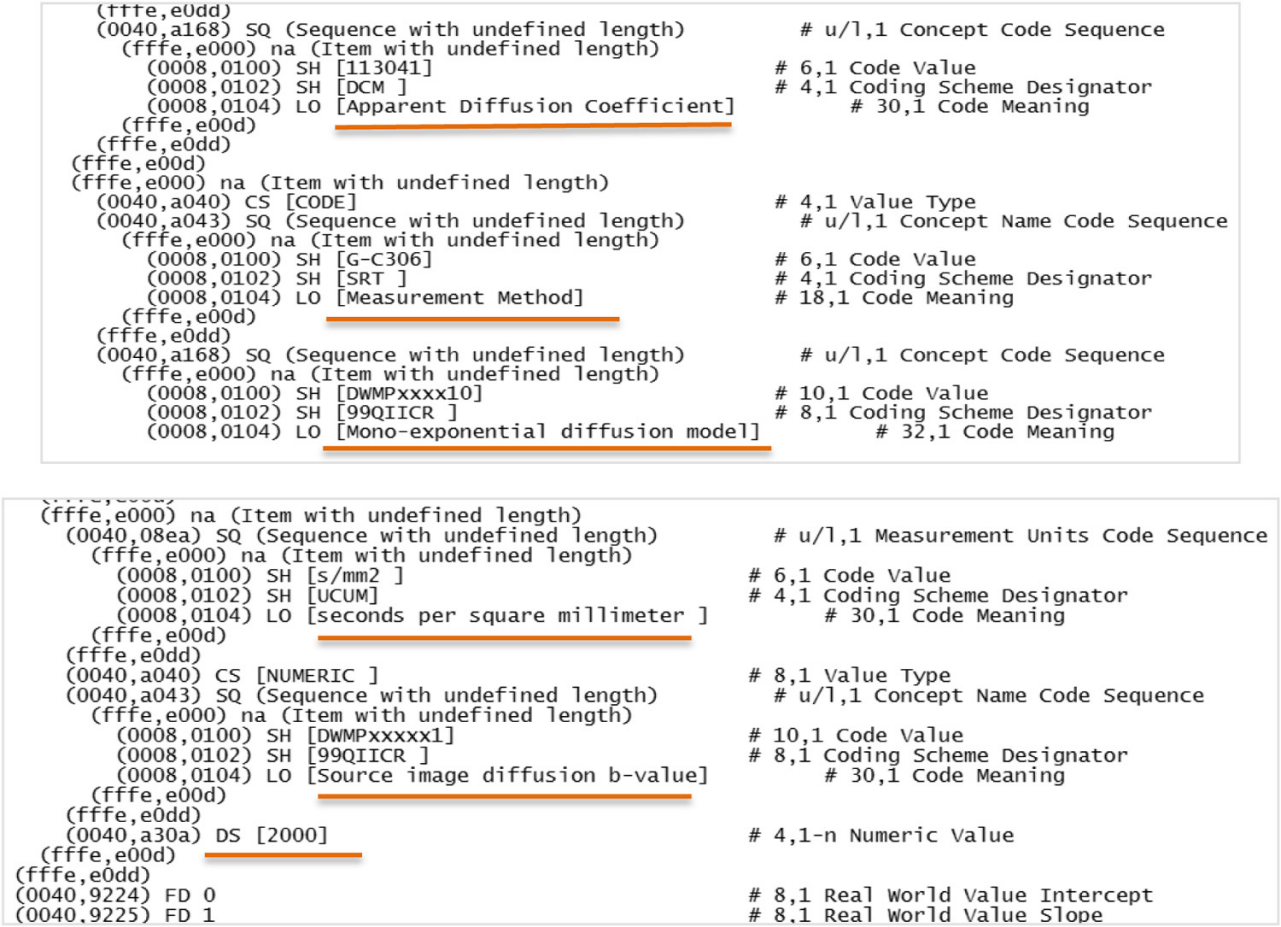
Example of critical ADC2 (S2) map metadata reflected in DICOM PM header generated by *dcmqi*.

None of the currently supported converter formats included those used by sites in this project (Table 2). The test ADC2 map DICOM converted by this tool properly reflected both source DWI geometry and all optional metadata specified by the standard (Appendix, Fig. 4). This optional metadata was not validated against source DWI DICOM (b-value) or analysis environment (algorithm.) Critical metadata regarding fit algorithm functions and parameters was not found (likely still missing from the standard). The object storage precision was not automatically discovered by MATLAB^®^ (*dicom-dictionary*), but the 32-bit floating-point volumetric images were recoverable from the “*dicominfo*” structure “data dump” field. In addition to MATLAB^®^, the test DICOM was successfully imported only by OsiriX and 3D Slicer (with *QuantitativeReporting* extension) and could not be parsed by iQ-View, IDL, ImageJ, 3D Slicer (without the extension), and KPacs viewers indicating limited back-compatibility for the new DICOM PM standard compared to legacy DICOM MR object.

## 4 Discussion

Overall high absolute numerical consistency was observed for ADC analysis results by participating sites with respect to the ground-truth ADC values of the PVP phantom. The processing-related variations were much smaller than systematic source DWI biases.^35^^,^^43^ Consistent with the results of the parent “ADC mapping” project for *in vivo* DWI,^27^ more complex fit models and multiparameter algorithms tended to show more variability with respect to the true ${\mathrm{ADC}}_{\mathrm{PVP}}$. Two-b fit ADC values have shown better consistency among in-house analysis tools compared to vendor SW, likely due to better control over DWI signal filtering options. ADC analysis tools provided by vendors had limited ability to parse cross-vendor DWI DICOM.

Lack of DWI DICOM standard compliance and interoperability among vendors apparently led to almost exclusive use of off-scanner processing by participating sites for multi-b ADC fitting. Higher relative deviations from the ground-truth ADC observed for multi-b fit ADC results (compared to two b-value model) were found to be directly related to the specifics of the applied fit algorithm. Analysis of variations for multi-b fit diffusion models provided strong evidence for the importance of capturing information on the diffusion model and parameters in the ADC map metadata. As a result of these findings, the corresponding coded terms and attributes, originally missing from PM DICOM standard, were added by submitting a correction proposal to the standard.^37^

Most of the project participants resorted to using in-house DICOM MR converter/parsers merged with their chosen programming environment to derive ADC maps in single-frame DICOM. The use of this legacy standard ensured general compatibility with many existing DICOM parsers and viewers. Among vendors, the ADC DICOM representation provided by ReadyView software was the closest to the PM standard.^22^ All sites found 16-bit precision sufficient for the storage of the derived ADC maps. None of the sites utilized the DICOM PM object representation,^23^ leading to lack of information critical to the interpretation of the fitting results. Specifically, information about the model used, b-values fitted, and units of measurement were either not present or not coded consistently in the standard attributes in the resulting ADC DICOM datasets. While supplying this missing metadata was possible with the ADC analysis for the phantom that provided ground-truth values, it would exceedingly complicate quantitative interpretation of *in vivo* results for multicenter trials.^27^ The DICOM PM object that provides a mechanism to capture most of this critical metadata in standard format is a relatively new addition to the standard and is not widely supported by the researchers, vendors, and tools, unlike DICOM MR. The notable exceptions of popular tools that support visualization of DICOM PMs are MATLAB^®^, OsiriX,^44^ and 3D Slicer^45^ (with *QuantitativeReporting* extension). To support conversion to and from DICOM PMs, the DICOM for Quantitative Imaging (*dcmqi*) library^25^^,^^38^ has been developed during this project.

Several limitations of the current *dcmqi* converter need to be addressed to facilitate wider adaptation of the technology by the QIN community. First, the tool does not provide mechanisms to curate optional metadata due to disconnect from the ADC analysis environment and output formats used by the project participants. Currently, sites would need to convert their volumetric image data into one of the inputs supported by *dcmqi* and provide original DWI DICOM as well as an ASCII metadata file to produce PM DICOM output. To mend the disconnect, ideally, the ADC analysis tools should provide means to record both DWI source-image information (containing b-values) and the numerical algorithm specific to analysis environments, and pass this information automatically as metadata to the converter. On the converter side, clear instructions should be developed demonstrating the process of conversion from the commonly used representations, such as legacy MR Storage object, into the DICOM PM storage.

Another limitation is that the only available converter output option of 32-bit multiframe cannot be viewed and parsed by most commercial and PACS viewers. Our results have shown that 12- to 16-bit ADC DICOM storage has provided sufficient ADC precision across the site implementations, which is in contrast to the currently implemented 32-bit storage format of the DICOM pixel data attribute supported by *dcmqi*. Since the original scanner source DWI is stored with 12- to 16-bit precision, less than $10^{3}$ ADC range is physically measurable for a single b-value weighting, which comfortably fits within 16-bit storage.^46^ In fact, our results show that even an order of magnitude change in ADC (PVP phantom) presents challenges for the current MR acquisition devices due to SNR limits at high b-values and CNR at low b-values. Considering these physical limitations and measurable range of tissue diffusivity values cited in literature [0.1 to 100 ($\times 10^{-3}\;{\mathrm{mm}}^{2}/s$)],^5^^,^^6^^,^^8^^,^^47^^,^^48^ implementation of 16-bit storage option would be adequate for ADC PM DICOM.

In summary, the use of DICOM PM standard for storage of quantitative diffusion images generated by multicenter studies and clinical trials is essential for interpretation and minimization of processing-related variabilities. Current deficiencies of ADC DICOM implementations based on nonstandard representations leave unexplained variability in centralized multisite meta-analysis. ADC units/scale, fit parameters/models, and source-image reference are critical attributes of PM DICOM for quantitative diffusion applications. Latency of new standard implementation by existing commercial DICOM parsers and PACS viewers poses a practical challenge. Promoting future uniform PM DICOM implementation across QIN sites, facilitated by *dcmqi* tools, can help leverage this technology for wider adoption by the quantitative imaging trial community and encourage timely implementation by MR and PACS vendors.

## Appendix: DICOM Header Check Results

Appendix shows screen-capture examples of the DICOM header check results for ADC maps based on DICOM MR and PM objects. Critical metadata missing in DICOM MR (Fig. 3) is captured by (underlined) PM attributes (Fig. 4).
